# Documenting Cervical Spine Injuries Following Negative MRI Findings: Clinical and Medico-Legal Overview of Dynamic Imaging

**DOI:** 10.7759/cureus.88121

**Published:** 2025-07-16

**Authors:** Leonard F Vernon, Adam Benn

**Affiliations:** 1 Clinical Sciences, Sherman College of Chiropractic, Spartanburg, USA; 2 Joint and Spine Chiropractic, Private Practice of Chiropractic, Camden, USA

**Keywords:** cervical instability, cervical lordosis, digital motion x-ray, dynamic imaging, mri, videofluoroscopy, whiplash, x-ray

## Abstract

Because of the lack of uniformity in describing injuries to the cervical spine following trauma, such as motor vehicle accidents, the Government of Quebec convened a multidisciplinary task force in the 1990s to attempt to standardize the terminology for the classification, management, and prognosis for such injuries. The term "whiplash-associated disorders" (WAD) was adopted and has become the universally recognized umbrella term for the myriad of symptoms caused by severe acceleration and deceleration forces applied to the head, craniocervical junction, and cervical spine following trauma. Obtaining an accurate diagnosis and prognosis for individuals with WAD is challenging, especially when magnetic resonance imaging and computed tomography findings are negative. This becomes more critical when litigation is involved. However, the often overlooked and underutilized imaging modality of video fluoroscopy/dynamic imaging is essential for the diagnosis of ligament instability, which can lead to hypolordosis of the cervical spine and has a long list of potential clinical consequences. The review highlights the usefulness of the cervical curve as a clinical indicator of the severity of vertebral column injuries.

## Introduction and background

The widely held belief that whiplash injuries are strictly soft tissue injuries is misleading and underestimates their complexity and potential severity [1]. This myth is concerning because it undermines the multifaceted nature of whiplash-associated disorders (WAD). It has led to the dismissal of legitimate pain complaints from patients with WAD as purely psychological, especially for cases with negative magnetic resonance imaging (MRI) findings [2]. The assumption that MRI is the gold standard for providing evidence of whiplash-induced injury is increasingly being challenged. Researchers have found that post-injury MRI findings are not associated with the diagnosis or prognosis of whiplash injuries. This means that the absence of observable structural abnormalities does not rule out pathology or the legitimacy of pain experienced by patients [3,4].

MRI has advantages for visualizing soft tissues. However, there are significant limitations that raise doubts about its reliability as an exclusive or definitive diagnostic tool for soft tissue injuries [5]. MRI is excellent for providing evidence of soft tissue injury when the focus is on pathologies such as intervertebral disc herniation. However, other injuries can occur in the cervical spine following motor vehicle collisions and are frequently associated with the straightening of the cervical curve. Ignoring this alignment alteration may result in overlooking injury severity.

Loss of the normal lordosis of the cervical spine is common in patients after motor vehicle accidents and manifests as the straightening or, in some cases, complete reversal of the curve [6]. Radiologists and treating clinicians often dismiss this finding as a muscle spasm; however, this is usually inaccurate [7].

This paper examines the clinical significance of cervical hypolordosis, the anatomy of the cervical spine, and the clinical significance of the surrounding ligamentous structures and the roles they play in the stability of the cervical spine. It also explores the value of imaging modalities other than MRI or CT for evaluating these structures and the mechanism underlying the association of injury to these structures and loss or reversal of the cervical curve. The loss or reversal of the cervical curve can indicate severe injuries and is associated with several symptoms. The aim of this review is to emphasize the usefulness of the cervical curve as a clinical indicator of injury severity and for prognostication [8].

Research on the sagittal alignment of the cervical spine initially focused on its correlation with the outcomes of surgical treatment for cervical myelopathy [9]. While the concept of sagittal spinal alignment has been extensively studied for the thoracolumbar spine since the 2000s, this research was only extended to the cervical spine in the 2010s. It is only in the last decade that the impact of cervical spinal alignment on spinal and general health has gained interest in the field of spinal deformity research [10].

**Figure 1:**
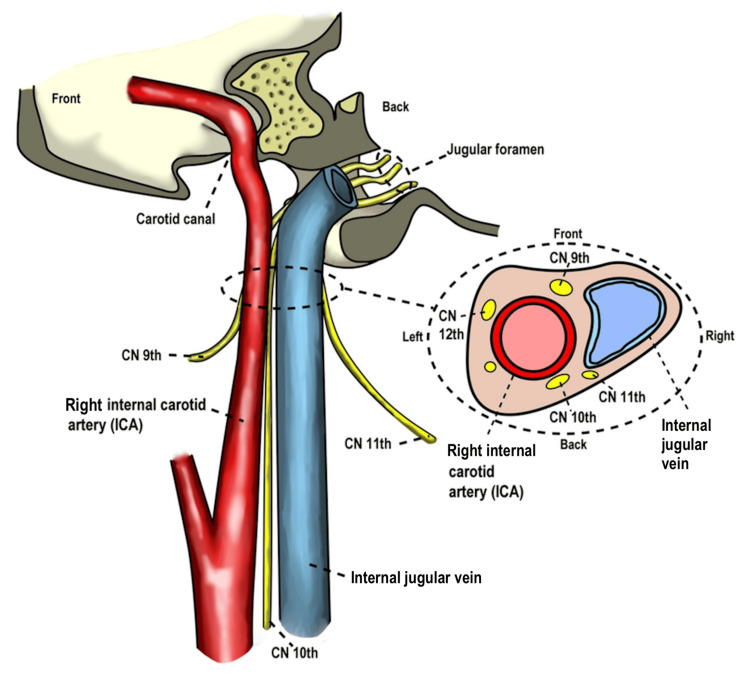
Carotid sheath The carotid sheath is composed of the common and internal carotid arteries, the internal jugular vein, and the vagus nerves. Source: Licensed from Shutterstock.

Loss of lordosis has been shown to cause compression, stretching, and encroachment of the structures in the carotid sheath, which is composed of the common and internal carotid arteries, internal jugular vein, and vagus nerves (Figure 1). It has been linked to a host of symptoms [11]. Symptoms associated with hypolordosis or kyphosis of the cervical spine include visual disturbances [12], fibromyalgia [13], headaches [14], and vertigo [15]. Other symptoms, such as numbness, tingling, confusion, cognitive deficits, trouble swallowing, and nausea/vomiting, have been reported in patients with a loss of cervical lordosis or increased kyphosis [16]. Normal cervical lordosis plays a critical role in maintaining the functional dynamics of neurovascular elements essential for proper neurological function. Research indicates that loss of normal spinal curvature can alter spinal canal dynamics and influence how cerebrospinal fluid circulates around the brain and spinal cord [17,18]. Some authors have linked these findings to the development of neurodegenerative diseases such as multiple sclerosis and amyotrophic lateral sclerosis [19-21]. The clinical significance of the loss of cervical lordosis should not be underestimated or dismissed as a benign finding; it should be thoroughly investigated to establish its cause and potential clinical implications.

## Review

Not a muscle spasm

An examination of the natural curvature of the cervical spine shows that it maintains its lordotic shape because of the wedge-shaped cervical vertebrae and the need to compensate for the kyphotic curvature of the thoracic spine [22]. Automobile accidents are among the most common causes of injuries that can disrupt the cervical curvature [23]. However, radiological findings of the loss of lordosis or the development of cervical kyphosis are associated with pain and disability [24,25], but they are often dismissed as indicative of benign muscle spasm. Several authors have supported the dismissal of these findings [26-29]. Rechtman et al. challenged this belief and stated that "flattening of a cervical lordosis should be evaluated carefully, especially in medico-legal problems, before being attributed to muscular spasm, as has been mentioned so commonly in radiological reports" [30]. Fedorchuk et al. reported that the results of their study "were in direct conflict with over 50 years of radiographic reports, physiological tests, and articles stating that loss or reversal of cervical lordosis is caused by cervical muscle spasms" [31]. Helliwell et al. reported similar findings [32]. The literature highlights that ligament injury is a major contributor to cervical hypolordosis [33,34], especially with a positive history of hyperflexion-hyperextension injuries such as those observed in WAD.

Thakar et al. reported that spinal ligamentous laxity supersedes the influence of spinal musculature in cervical spine injury [35]. However, Hass et al. indicated that the posterior muscles are bulkier than the anterior muscles, and muscle spasms should cause hyperlordosis due to the extension of the head and neck rather than cervical spine straightening or kyphosis. They emphasized "that cervical spine muscle spasm or hypertonicity is a co-variable of the reaction to an injury and is not a cause of altered cervical sagittal alignment" [36]. Other reports of severe ligament injuries in the absence of radiographic findings have been published, including that by Scher, which states that "ligamentous damage can be present in the cervical spine after injury without radiographic evidence of fracture or dislocation and is often represented by loss of the cervical lordosis. If the posterior ligament complex is completely disrupted with resultant instability, the patient is at risk of developing late complications. Instability may persist indefinitely, as healing, sufficient to restore the original ligamentous strength, may not take place. Persistent instability may produce pain or progressive neurological damage. Late intervertebral displacement and late vertebral deformity are other potential complications" [37].

Hypolordosis is simply the visual result of an often overlooked underlying ligamentous injury. This injury can cause instability of the craniocervical junction and has several effects on surrounding structures with short- and long-term clinical implications. To better appreciate this, we present a brief overview of cervical spine anatomy.

Cervical spine anatomy

The vertebral column usually consists of 33 vertebrae divided into the cervical, thoracic, lumbar, sacral, and coccygeal. Its primary function is to protect the spinal cord while facilitating movement in various planes, such as flexion and rotation. The cervical spine, which comprises seven vertebrae, is the most complicated articular system in the body [38] and the most significant of all the spinal regions. The vertebrae of the cervical spine contain a much wider spinal canal than those of the other regions due to their proximity to the head and their role in protecting the upper spinal cord and various neurological, arterial, and venous structures, such as the vertebral arteries, that contribute to the posterior circulation of the brain. From a biomechanical standpoint, this region is responsible for several sophisticated head and neck movements, and normal function requires that all movements be performed without damaging the spinal cord, vascular supply, or millions of nerve fibers [39]. This makes it a region of special interest in the context of trauma [40].

The cervical spine comprises seven vertebrae; however, it is clinically divided into two groups according to their peculiarities. The superior cervical group comprises the posterior inferior aspect of the skull at C0 (occiput), C1 (atlas), and C2 (axis), while the inferior cervical group comprises C3 to C7 [41]. The superior group, which is essential for maintaining craniocervical stability, is known as the craniocervical junction (CCJ). The CCJ comprises two distinct joints: the atlanto-occipital (AO) joint, which consists of the skull (occiput) resting on the first cervical vertebra (atlas), and the atlantoaxial (AA) joint, where the atlas connects with the axis. Several ligaments, muscles, small joints called facet joints, and occipital condyles (OC) hold the head and allow it to move in various directions, including flexion, extension, rotation, and lateral tilting. The atlas and axis have unique anatomical features that are not found in cervical vertebrae C3-C7, which are relatively uniform in shape and appearance.

**Figure 2:**
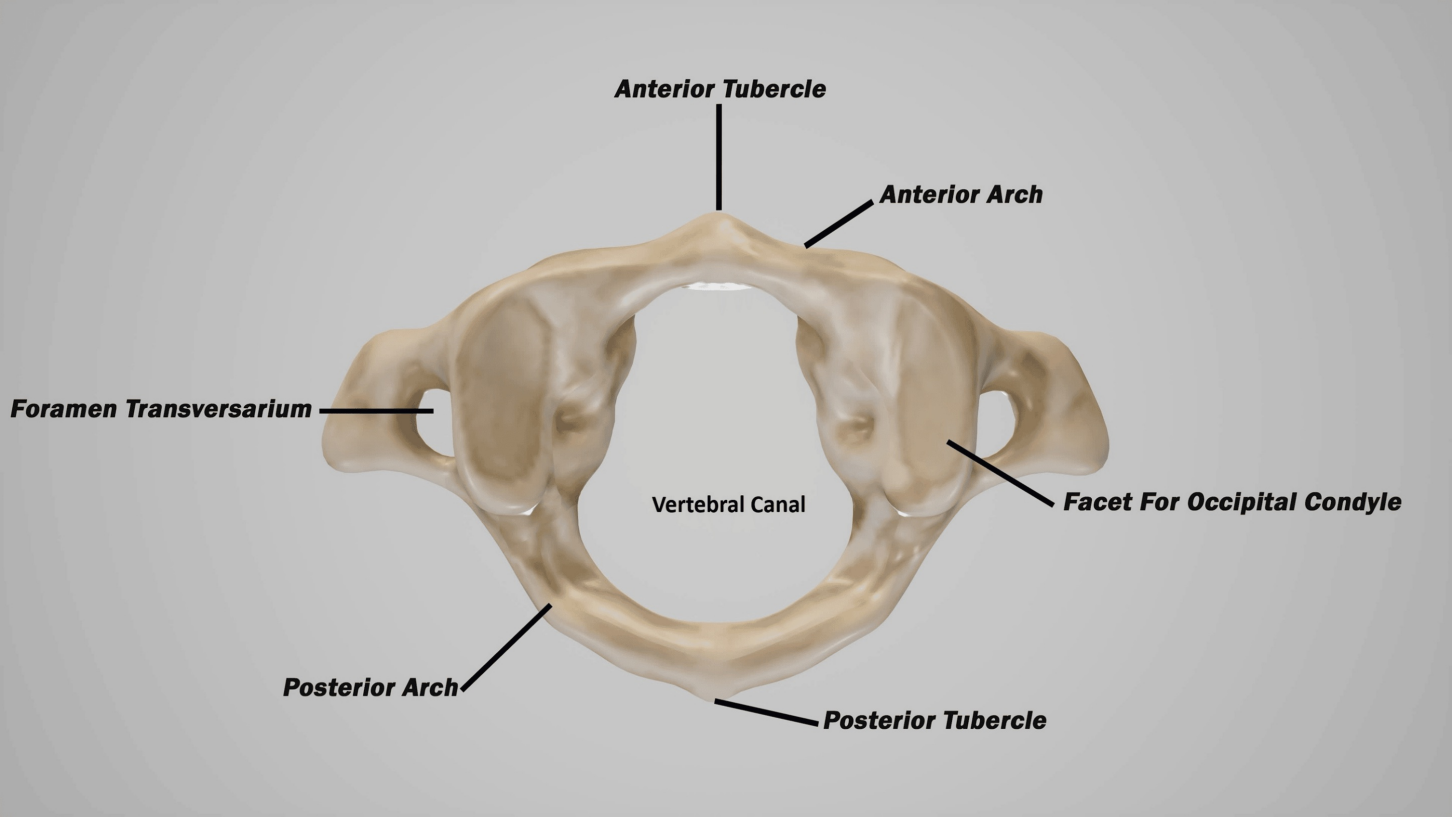
The C1 vertebra (atlas) The C1 vertebra, also known as the Atlas, named after the Titan Atlas from Greek mythology, who bore the weight of the world on his shoulders, supports the head. It has a central opening for the passage of the spinal cord, as well as foramina in its transverse processes for the passage of the vertebral artery. Source: Licensed from Shutterstock.

The atlas vertebra (C1) lacks a vertebral body and a spinous process, unlike the other cervical vertebrae, and it is considered atypical. Instead of a vertebral body, the atlas comprises anterior and posterior arches joined by a lateral mass on each side. Each lateral mass bears a kidney-shaped superior articular facet that articulates with the occiput above it via the OC to form the AO joint. Each facet projects a transverse process laterally that contains foramina, the significance of which is discussed later in this paper. The anterior and posterior arches and the two lateral masses form a central ring-like anatomical space called the vertebral canal, which provides a passage for the spinal cord (Figure 2) [42].

**Figure 3:**
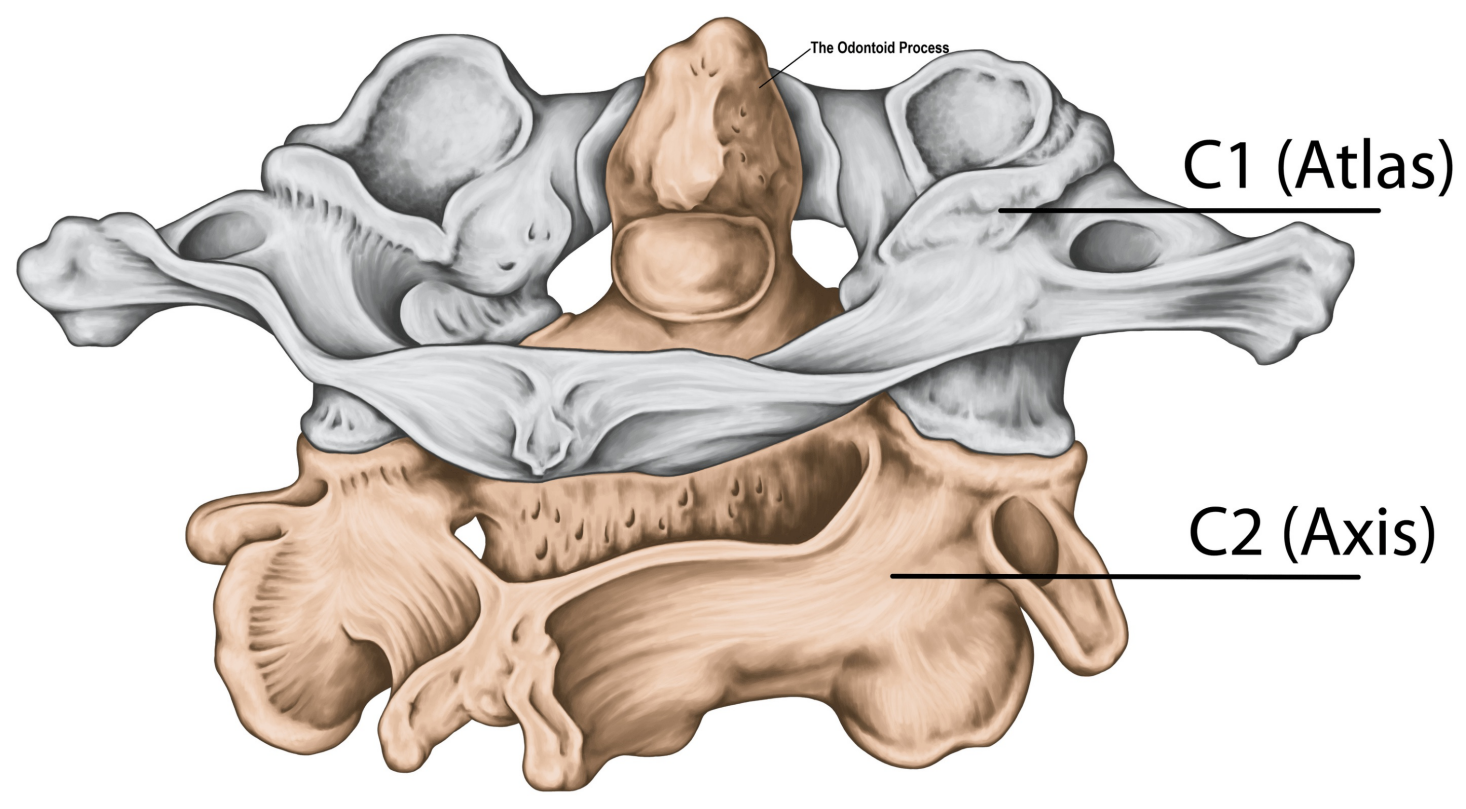
The C2 vertebra (axis) One of the most obvious hallmark features of the atlas is a bony projection known as the odontoid process (dens). The odontoid process acts as a stable pivot around which the atlas and head rotate. Source: Licensed from Shutterstock.

The C1 vertebra has inferior facets that articulate with the C2 vertebra (axis) below. The axis forms a pivot for the rotation of the first cervical vertebra (Figure 3). Unlike the C1 vertebra, the axis has a vertebral body and a spinous process, making it similar to other cervical vertebrae, but it has peculiarities. One of the most obvious of these is the hallmark feature of the odontoid process (the dens), which is a bony projection extending cranially from the vertebral body.

The odontoid process evolves from the body of C1, which separates during early fetal life and fuses with the body of the axis [43]. The odontoid process serves an important function in the movement of the head. It acts as a stable pivot around which the atlas and head rotate and is the principal attachment point for the ligaments stabilizing the atlantoaxial junction [44]. There is no intervertebral disc between C1 and C2, but the C2 vertebra has transverse processes with foramina like the other vertebrae of the spine [45,46].

Vascular and neurological elements of the cervical spine

Cervical stability is best demonstrated when the plethora of nerves and vascular structures that travel around and through the cervical spine are understood. For this paper, we limited our review to the most significant vascular and neurological structures of the CCJ and the clinical significance of instability in this region.

**Figure 4:**
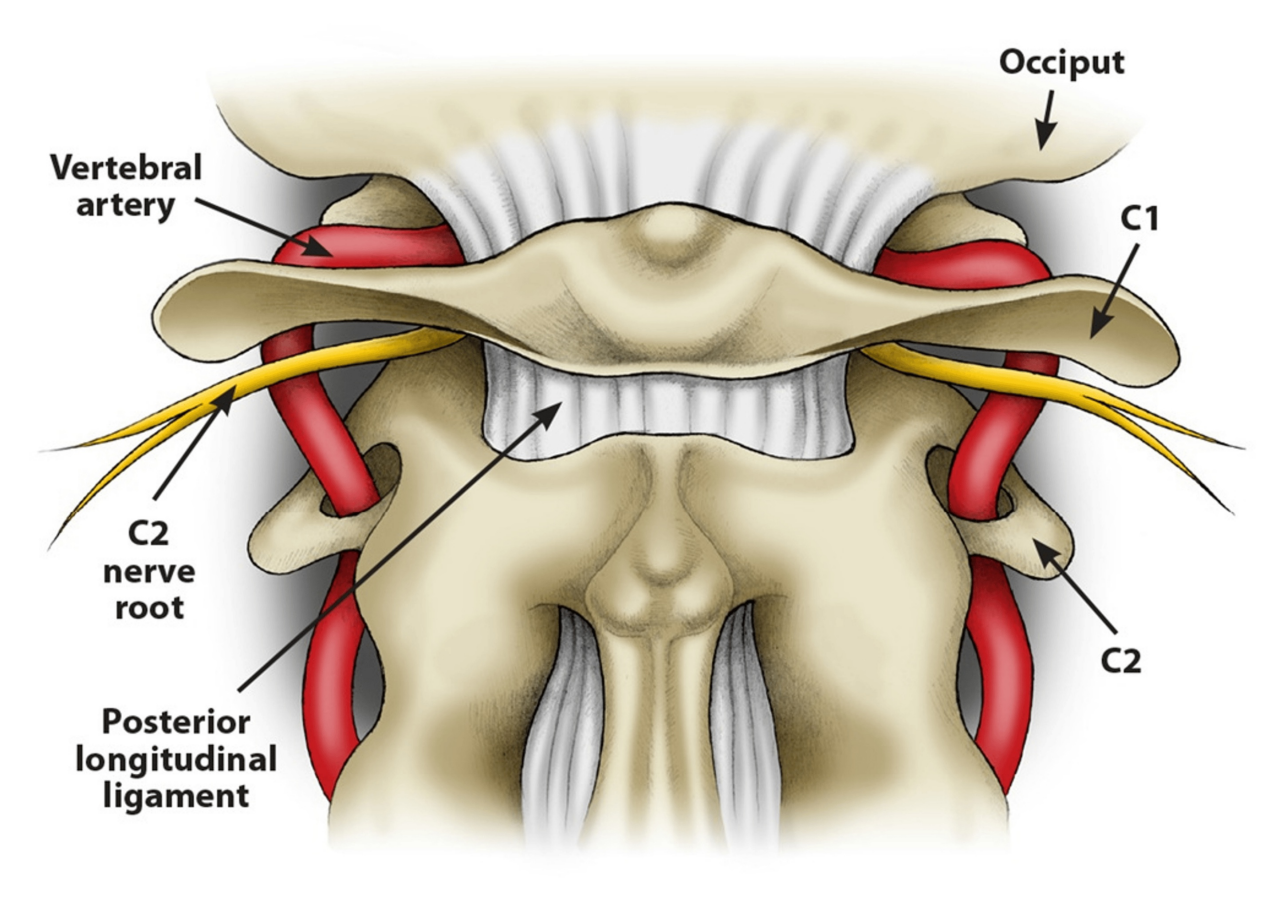
C1 and C2 articulation with the vertebral artery passing through the foramina in the transverse processes of each vertebra Anterior to the posterior arch of C1 and posterior to each articular process, there is a groove where the artery runs. Source: Used with permission from Caring Medical, Ross Hauser, MD.

The cervical vertebrae have one thing in common: they all have a transverse process on each side of the vertebral body except for C1, which lacks a body. Each transverse process has a foramen through which vital nerves and blood vessels pass, including one of the major suppliers of blood to the brain (20%) and the vertebral arteries (Figure 4).

**Figure 5:**
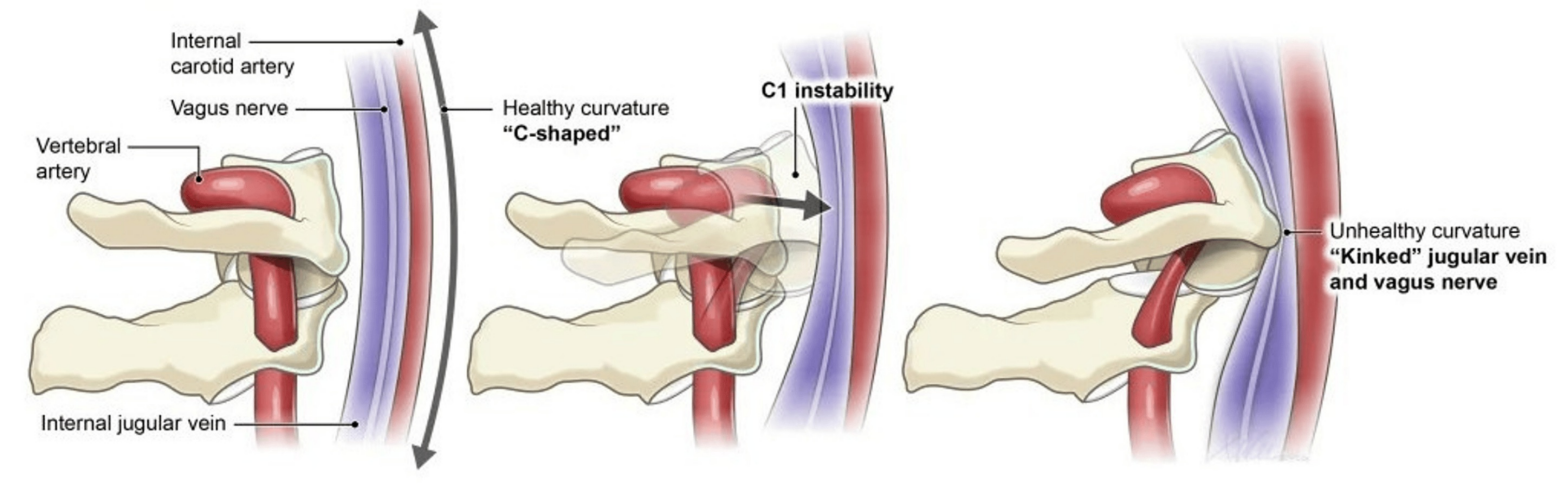
Compression of the carotid sheath Compression of the carotid sheath and kinking of the vertebral artery can occur with C1 instability. Source: Used with permission from Caring Medical, Ross Hauser, MD.

Anterior to the posterior arch of C1 and posterior to each superior articular process, there is a groove where the artery runs [47]. It is at these levels, C1 and C2, that the vertebral artery is vulnerable to trauma [48,49]. Goel and Cacciola [50] described the path of the vertebral arteries as serpentine. The other 80% of the blood is carried to the brain by the internal carotid artery, which sits in front of the transverse processes of C1 (atlas) and C2 (axis). It is vulnerable to compression in the presence of cervical spine instability (Figure 5) [49,51].

In addition to vascular compression, CCJ instability can be associated with compression of the brain, brainstem, and cranial nerves [52]. Clinically, compression of the cranial nerves often results in symptoms [53,54]. They emerge directly from the brain or brainstem and are a set of 12 nerves that are both motor and sensory. They are responsible for several physiological processes, including vision, olfaction, audition, and complex facial movements. Unlike spinal nerves, which emanate from segments of the spinal cord, cranial nerves are directly connected to specific brain regions. They form a crucial interface for the transmission of specialized sensory and motor signals [55]. Cranial nerve compression can manifest in various symptoms depending on which nerve is affected and the extent of compression. Common symptoms include pain, numbness, tingling, and weakness of the face, as well as vision changes, double vision, and hearing problems [56].

**Figure 6:**
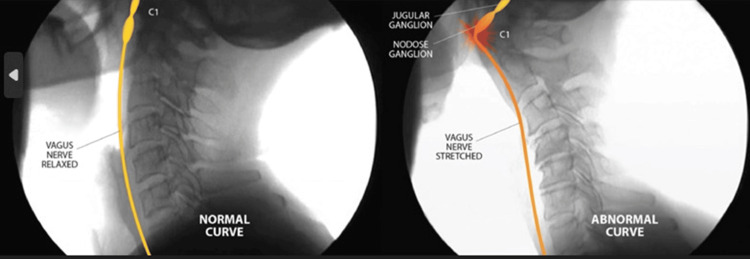
Reversal of cervical spine affecting the vagus nerve (CN X) In the normal, healthy lordotic curve, the vagus nerve does not encounter any resistance. In an abnormal curve caused by instability due to ligament incompetence, the vagus nerve is under stretch and compression. Source: Used with permission from Caring Medical, Ross Hauser, MD.

The involvement of cranial nerve X (CN X) can manifest in several unrelated symptoms. It is known as the vagus nerve, derived from the Latin word for "wandering, straying." Within the carotid sheath, the vagus nerve enters the brain through the jugular foramen. The vagus nerve path is directed into the foramen when a person has a normal cervical lordotic curve. However, it has to make a 90° turn and kinks at the level of the C1 (atlas) vertebra with changes in the cervical curve due to ligamentous cervical instability (Figure 6).

**Figure 7:**
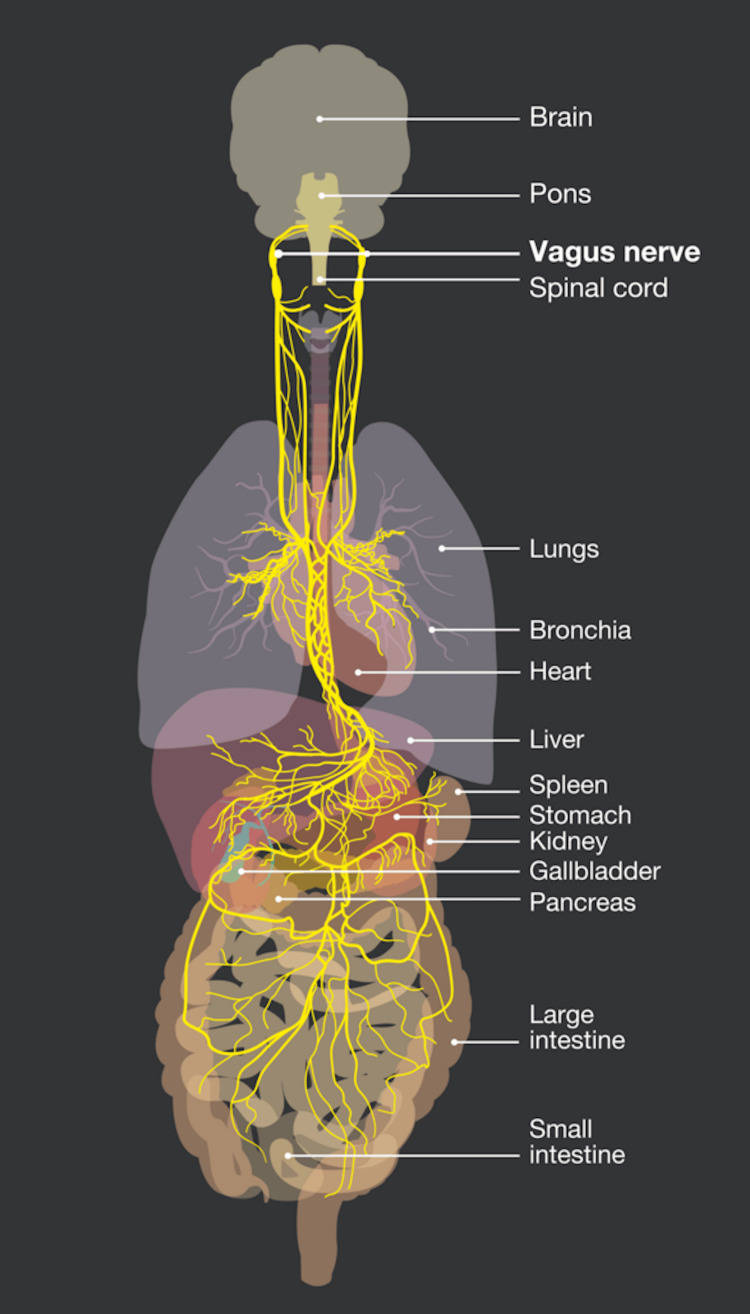
Distribution of the vagus nerve The vagus nerve follows a complex course throughout the body and innervates multiple organs. It originates in the brain stem and terminates in the splenic flexure of the colon. Source: Licensed from Alex Kock/Science Photo Library.

The vagus nerve follows a complex course throughout the body and innervates multiple organs. It originates in the brainstem and terminates in the splenic flexure of the colon (Figure 7) [57]. Efferent (motor) parasympathetic fibers from the vagus, which represent approximately 20% of the nerve, control several functions of the cardiopulmonary and gastrointestinal systems, including digestion, satiety, respiration, blood pressure, and heart rate. Vagus nerve compression has been linked to the symptoms of dysautonomia [58].

**Figure 8:**
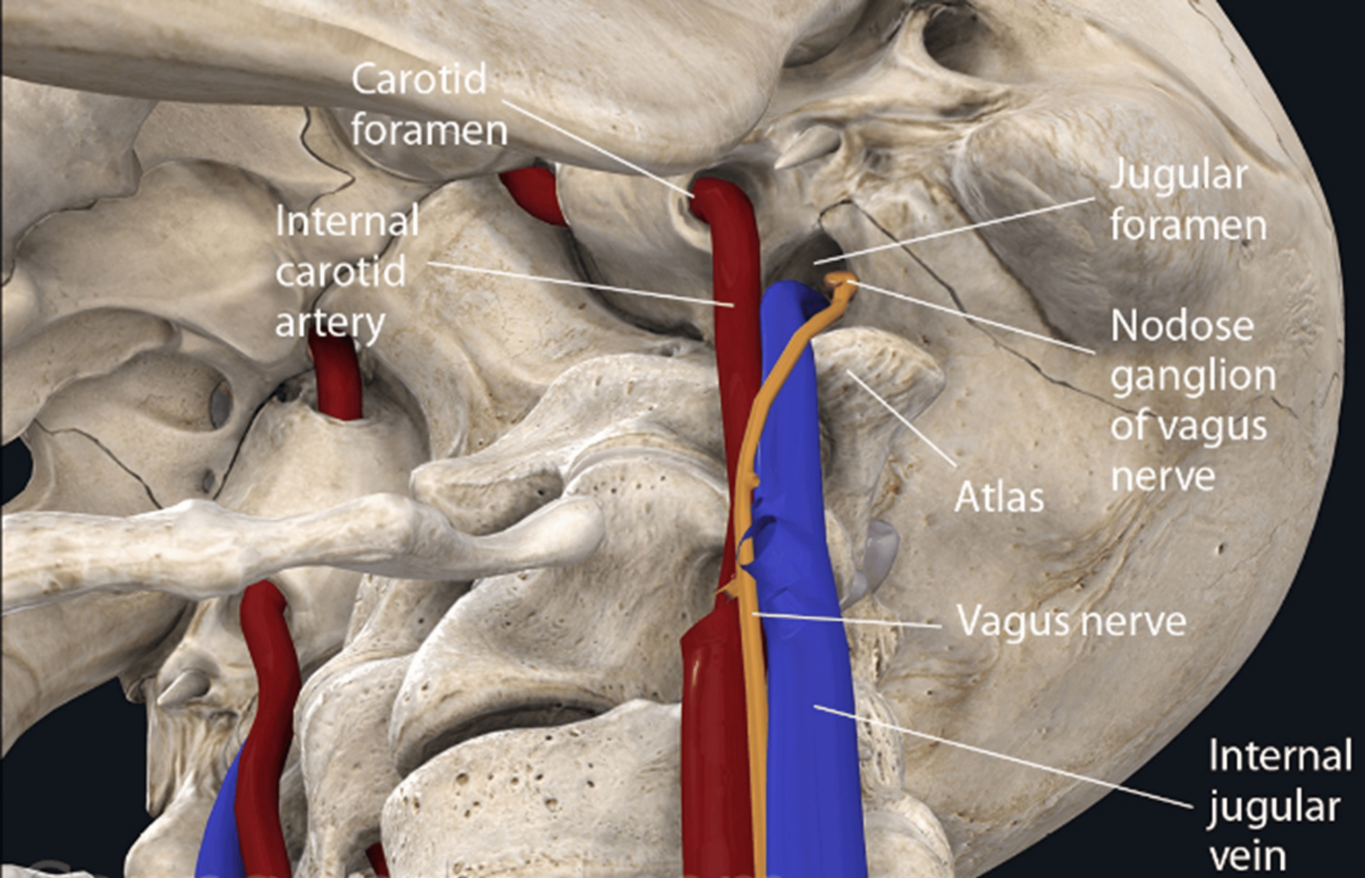
Afferent cell bodies of the digestive tract are located in the nodose ganglion Instability of the cervical spine can cause organic symptoms, given that the afferent cell bodies of the nerve are located in the nodose ganglion, which is located in front of the transverse process of C1. Source: Used with permission from Caring Medical, Ross Hauser, MD.

Any interruption in its function may have severe consequences, including gastroesophageal reflux disease, heart failure, respiratory control failure, gastroparesis, vasovagal syncope, and chronic pain [59]. General visceral afferent (sensory) fibers carry sensory information from the thoracic and abdominal viscera, the aortic body, and the aortic arch while innervating numerous skeletal muscles [60]. An example of how the upper cervical spine and ligamentous instability can affect vagus nerve function can be appreciated by considering that the afferent cell bodies from the digestive tract lie in the nodose ganglion, which is in front of the transverse processes of the C1 vertebra (Figure 8).

The vagus nerve cell bodies in the jugular ganglion, which provide somatic and visceral sensory neurons innervating the external auditory canal, tympanic membrane, cranial meninges, pharynx, and larynx, are also in proximity [61]. Matsui et al. examined the occurrence of "pain all over" in patients with whiplash. They reported that a "considerable number of patients with WADs report variable and indefinite symptoms involving the entire body, despite the lack of evidence of direct injuries to organs other than the neck" [62].

The etiology of most changes in the cervical curve involves stretching of the posterior ligament complex of the cervical spine, especially the capsular ligaments, which causes anterolisthesis of the vertebrae and potential stretching and compression of the carotid sheath and its contents, including the vagus nerves [63]. Several ligaments that aid in the stability of the CCJ can become injured in trauma, such as a rear-end collision, and cause cervical spine instability and clinical conditions such as loss of cervical lordosis and symptoms associated with this.

Ligaments involved in maintaining cervical stability

The integrity of the cervical ligaments plays a critical role in maintaining the cervical lordotic curve and the stability of this segment of the vertebral column. However, this overreliance on ligamentous structures for stability in this segment of the vertebral column, coupled with the cervical spine sacrificing stability for mobility [64], makes it extremely prone to injuries [65].

**Figure 9:**
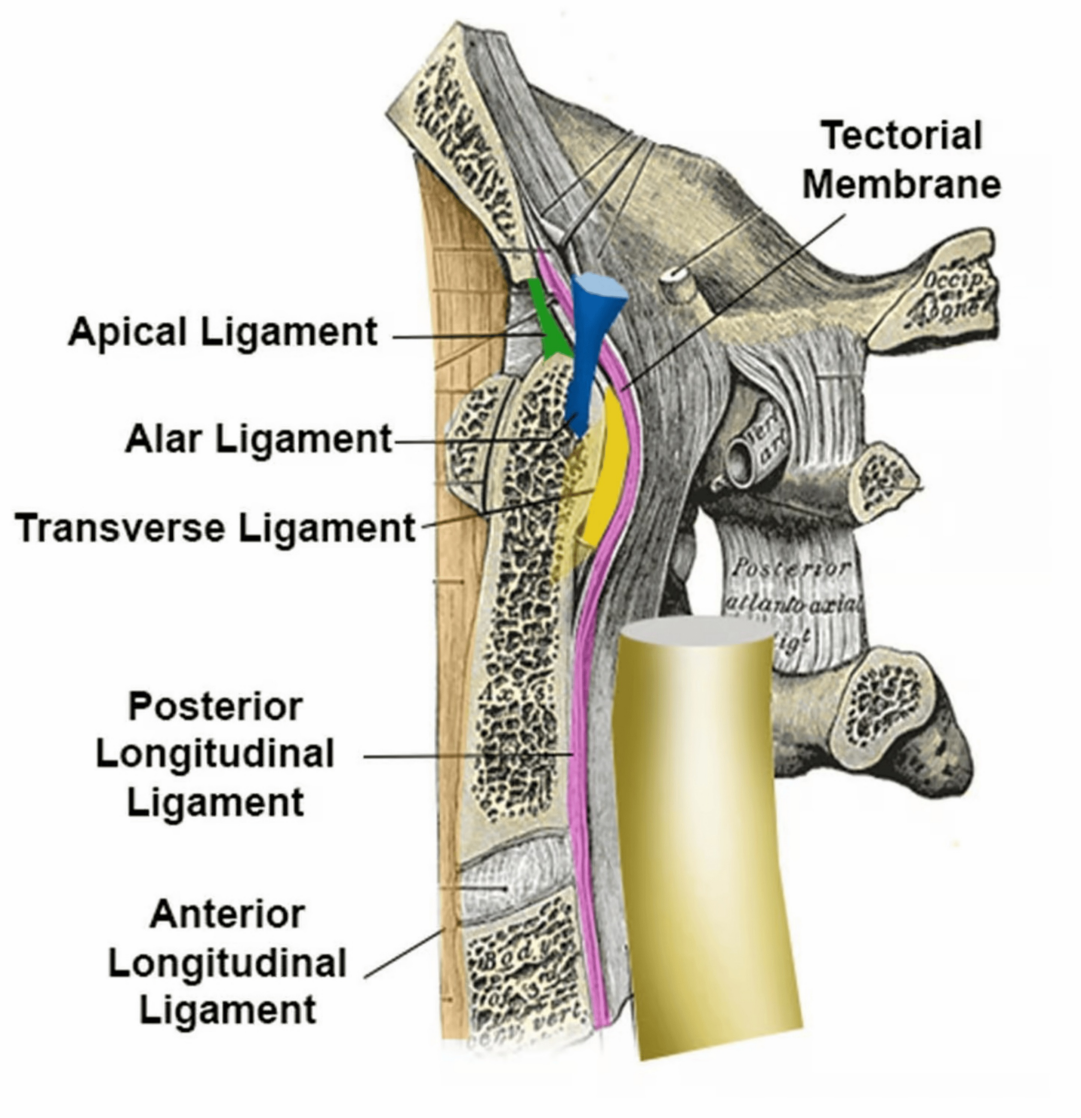
Cervical capsular ligaments Limitations in the functionality of the cervical ligaments due to whiplash can increase the laxity of the cervical capsular ligaments. Research has indicated that loss of tensile strength in these ligaments corresponds to the development of instability and abnormal curvature. Source: Licensed from Shutterstock.

The limitations in the functionality of the cervical ligaments due to trauma, such as whiplash injuries, can significantly increase the laxity of the cervical capsular ligaments. A decrease in ligament strength directly impacts spinal stability and alignment [34,66]. The nuchal ligament, anterior longitudinal ligament (ALL), posterior longitudinal ligament (PLL), tectorial membrane, alar ligaments, and transverse ligaments are the most frequent ligaments implicated in cervical instability and loss of lordotic curvature following trauma. Each of these structures has its function in aiding the stabilization of the joint, and minor injuries to any of these structures, such as stretching of the ligament, can result in joint instability. This can lead to a multitude of musculoskeletal and non-musculoskeletal symptoms (Figure 9) [67].

The nuchal ligament, which is positioned posteriorly, is vital for maintaining lordotic curvature and cervical stability. Injury to the nuchal ligament can lead to progressive malalignment and instability of the cervical spine and contribute to the loss of normal curvature [68]. The tectorial membrane, which connects the occiput to the atlas, acts as a significant stabilizer of the craniovertebral junction. Injury to the tectorial membrane has been associated with loss of mobility and increased mechanical stress in cases of cervical spine trauma [69].

The ALL extends from the base of the skull and runs the length of the entire spine. In the cervical spine, it stretches to prevent excessive extension. The PLL extends from C2, along the back of the neck, to the sacrum. It is a crucial ligament for spinal stability and function, primarily for resisting flexion and preventing posterior disc herniation. Compromised integrity of the ALL and PLL due to trauma has been linked to abnormal spinal alignment. Research has indicated that the loss of tensile strength in these ligaments corresponds to the development of instability and abnormal curvature [70].

The transverse ligament is the primary stabilizing ligamentous structure of the CCJ [71]. It is the strongest and thickest ligament, found posterior to the odontoid process, and it attaches bilaterally to the lateral process of the atlas. It has been described as a seatbelt for the dens [72]. The alar ligaments are the next most important ligaments for stabilizing the CCJ. They originate from the posterolateral side of the odontoid process and extend upward and laterally to attach to the medial surfaces of the OC. They restrain rotation and restrict side-to-side motion, and they attach the dens to the skull.

**Figure 10:**
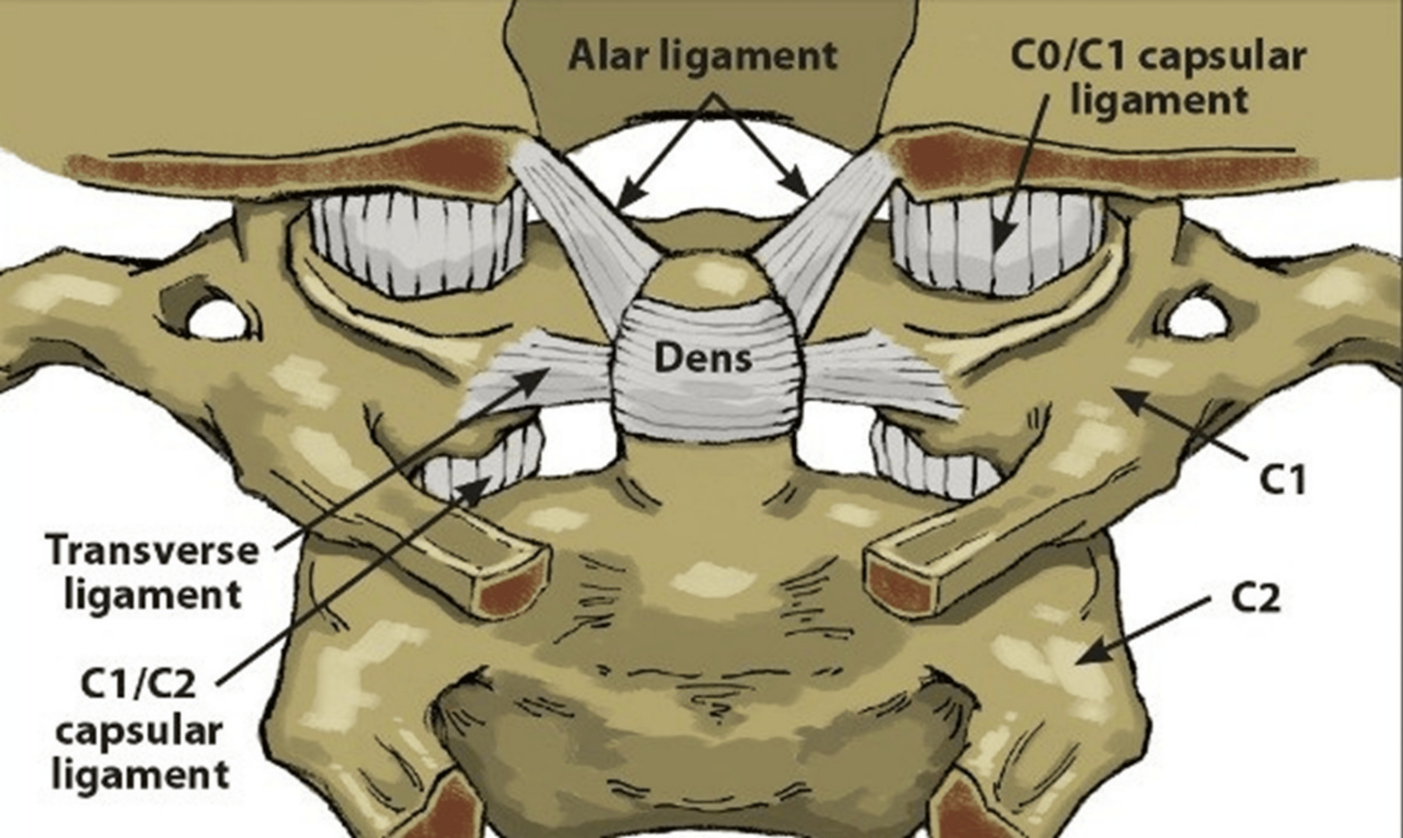
Alar ligaments extend from the posterior lateral aspect of the dens Along with the posterior longitudinal ligament (PLL), the alar ligaments prevent the anterior slippage of the head onto the cervical vertebrae. Source: Used with permission from Caring Medical, Ross Hauser, MD.

The alar ligaments extend from the posterior lateral aspect of the dens of the C2 vertebra and extend bilaterally upward and outward in a "V" shape, inserting into the medial aspect of the OC. Along with the PPL, the alar ligament prevents the anterior slippage of the head onto the cervical vertebrae. Dvorak et al. stated that the primary role of the alar ligaments is to contribute to craniovertebral stability (Figure 10) [73].

Diagnosis of cervical instability

Radiographic evaluation is crucial for identifying cervical spinal instability. Lateral X-rays, especially in flexion and extension views, are the standard initial assessments for visualizing the alignment of the cervical vertebrae and assessing subluxation [74]. Despite normal initial radiographic findings, injuries to CCJ ligaments can result in significant instability and neurological deficits if not accurately diagnosed [75].

MRI can play a role in diagnosing cervical instability when ligamentous injuries are suspected. However, its sensitivity for detecting ligamentous injury is estimated to be no more than 75%. This often leads to missed diagnoses of cervical instability, especially when subtle ligamentous damage occurs without significant accompanying structural changes visible on MRI scans [76,77]. Further complicating the diagnosis is the fact that injuries to critical ligaments, such as the tectorial membrane and alar ligaments, may not always be apparent on MRI. Fiester et al. reported that injuries to major ligaments in acute cases can yield false negatives during MRI evaluation [69]. Uhrenholt et al. reported that instability is a diagnosis of abnormal function. The condition is typically not identifiable on static radiographs or other conventional imaging techniques, such as MRI and CT, and is prone to underdiagnosis [78]. Jannsen et al. demonstrated the limitations of traditional imaging approaches in identifying subtle yet significant cervical instability [79], while Katz et al. indicated that cervical instability due to laxity of capsular ligaments or dynamic spinal cord compression during flexion/extension may evade detection on static imaging [80]. Yeo et al. reported that approximately 30% of patients with cervical trauma experience a restricted range of motion due to acute neck pain, causing standard flexion-extension radiographs to yield false-negative results for detecting instability [81]. In summary, the identification of cervical ligament instability using MRI has several challenges, especially because of the limitations of the modality in accurately visualizing soft tissue injuries when changes are subtle. Mantripragada reported that the complexity of the cervical spine presents interpretational challenges for radiologists and can often lead to missed diagnoses [82]. It should be noted that while standard cervical MRI scans do not adequately visualize the small ligaments of the CCJ region, these ligaments and other soft tissues of the CCJ region can be directly visualized using special high-resolution MRI techniques, such as the promising new technology for assessing the integrity of this region dynamic MRI [83]. While offering real-time visualization of the cervical spine during neck flexion and extension and identifying cervical spine instability and assessing the integrity of this region, the use of dynamic MRI as a routine screening is limited by the availability of the test and the high cost of the procedure [84], whereas the use of dynamic open-mouth imaging such as DMX is quite cost-effective and involves a relatively low radiation dosage.

In addition to the anatomical challenges in identifying cervical spine ligament injuries using standard static imaging, gravity- and body weight-related issues persist. Standard static imaging procedures are performed with patients in the supine position. Imaging in the supine position is useful for obtaining clear and controlled views of the cervical spine, but it is limited in accurately assessing cervical instability. The effects of gravity and body mechanics are altered in the supine position and may mask certain instabilities. For instance, Dave et al. suggested that body weight and gravity can perpendicularly compress unstable vertebrae when patients are lying supine. This can limit the visual detection of certain forms of instability, such as distraction instability [85]. In consequence, stability may be overestimated because the vertebrae are stabilized against one another due to the patient's body weight. Supine static imaging also cannot capture dynamic spinal motion. For example, traditional MRI scanning (usually performed in a neutral position) may not reveal the full extent of cervical lordosis or alignment changes that occur during motion. Karabağ and İplikçioğlu discussed this limitation, highlighting that traditional imaging does not replicate the dynamic conditions of the cervical spine exerted during daily activities [86]. Therefore, supine imaging alone may not adequately account for functional outcomes related to cervical instability. Supplemental imaging modalities are essential to prevent missed diagnoses and ensure the appropriate management of patients with suspected cervical instability. Imaging sequences that can account for and mitigate movement limitations in acute patients, such as those with WAD, are essential.

Video fluoroscopy is a supplemental imaging modality that has been valuable for detecting cervical instability, especially when other imaging methods may be inadequate. Video fluoroscopy provides a dynamic assessment of the cervical spine during motion, which helps clinicians understand how the cervical spine behaves in real-life scenarios rather than in controlled conditions that do not replicate the everyday movements essential for identifying subtle forms of instability.

Videofluoroscopy is underutilized in clinical practice relative to more established modalities, such as MRI and CT, despite its established utility. Moskopp emphasized the potential for automated image analysis in videofluoroscopy to dynamically enhance the assessment of cervical stability, which could improve diagnostic precision [87]. This idea may not be far off considering the advancements in artificial intelligence (AI). Integrating videofluoroscopy into clinical assessment protocols represents a valuable opportunity to improve diagnostic capabilities for detecting cervical instability, given the limitations of traditional imaging modalities.

**Figure 11:**
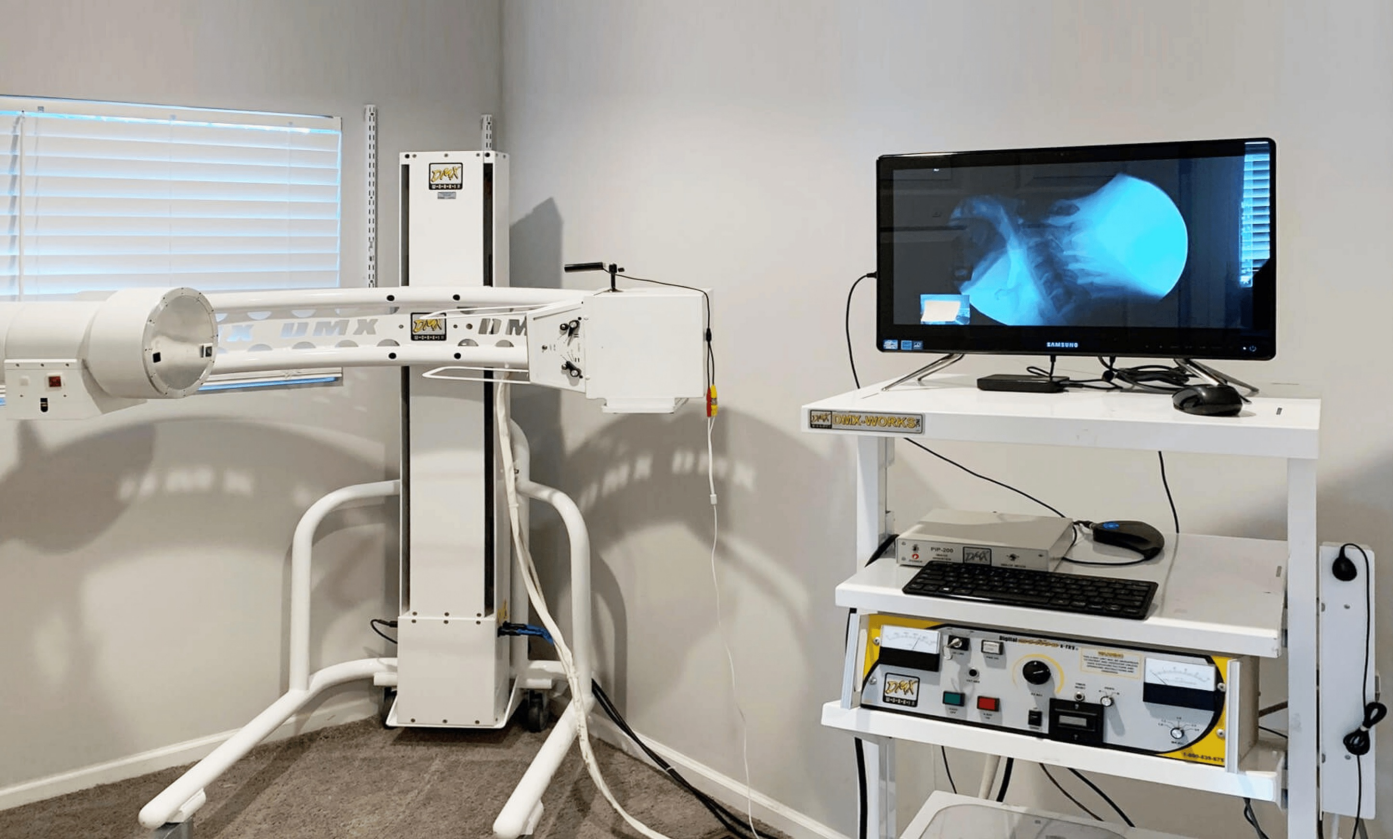
DMX Digital Motion X-ray equipment The equipment emits X-rays in real time to capture the cervical spine while the patient is put through various ranges of motion. Source: Used with permission from DMX Imaging, LLC (Palm Harbor, FL, USA).

Videofluoroscopy, also known as dynamic fluoroscopy, is performed in an office setting by positioning the patient upright or seated to allow for natural cervical spine movement. Proper positioning is crucial because it simulates normal gravitational forces experienced during everyday activities. Digital Motion X-Ray® (DMX Imaging, LLC, Palm Harbor, FL, USA), which is widely used in private office settings, records 30 images per second of continuous X-rays and captures the active range of motion. It provides dynamic four-dimensional visualization of the integrity of the ligaments of the upper cervical spine, allowing for the assessment of both static and dynamic parameters of vertebral alignment (Figure 11) [88].

The equipment emits X-rays in real time to capture the cervical spine while the patient places their neck in various positions, including flexion, extension, and lateral bending. The fluoroscopic camera continuously records these movements for real-time visualization of the relationships between the cervical vertebrae under various stressors. Because the radiation is pulsed, rather than constant stream technology, and only uses a 2-3 kilovoltage peak (kVp) versus the 80 kVp used for a typical plain cervical X-ray, the total radiation dose for a five VF motion study is approximately equivalent to the dose used for a seven-view cervical Davis series [89].

The ligaments are not directly visible, but the procedure allows clinicians to observe the cervical spine alignment and detect abnormal displacements or excessive motion between vertebrae, thus inferring ligamentous injury, especially when symptoms begin shortly after trauma. MRI can detect some ligament injuries, such as complete tears, but the type of stretching injury seen on VF usually does not correlate with abnormal CT or MRI findings [90].

**Figure 12:**
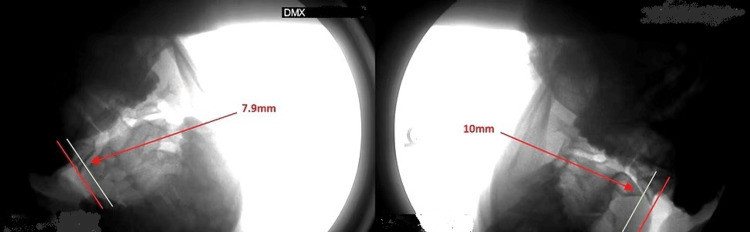
C1 and C2 lateral translation Lateral overhang indicates an interruption in the integrity of the alar ligaments. Source: Used with permission from DMX Imaging, LLC (Palm Harbor, FL, USA).

In addition to real-time visualization, a recording was obtained, and the images were reviewed frame-by-frame to assess the range of motion and any kinematic abnormalities of the cervical spine. This analysis is critical because it provides a detailed understanding of cervical segment functionality and stability under dynamic conditions (Figure 12).

Medico-legal use of dynamic imaging

Cervical trauma injuries frequently result from motor vehicle accidents, and these cases sometimes end up in court. However, convincing a jury of the severity of the injury is essential for the recovery of damages. The challenge becomes more difficult when jurors tend to rely on common misconceptions about fraud in personal injury claims, including claims related to whiplash. These misconceptions often overshadow legitimate cases [91]. Jurors may find it difficult to believe claims of significant suffering; widely accepted tests, such as MRI, are unable to demonstrate injury, given that soft tissue injuries do not manifest in physical signs. This finding aligns with those of Liberman et al., who demonstrated that jurors are more likely to be swayed by compelling evidence that effectively conveys the severity of an injury, often favoring the visualization of injuries when determining credibility and damages [92]. Ruva et al. found that the manner in which evidence is presented in court significantly influences the perceptions of jurors, even when they may initially harbor skepticism. This can significantly impact their decisions [93]. It allows for the identification of conditions that may not be evident in static assessments [94], due to its ability to visualize the cervical spine in motion, making it an excellent courtroom tool.

Discussion

Cervical lordosis refers to the natural inward curvature of the cervical spine, which plays a crucial role in maintaining overall spinal alignment and balance. The loss of the lordotic curve, often leading to a straight or kyphotic posture, can have significant clinical implications. Various studies demonstrate the correlation between loss of cervical lordosis and neck pain, decreased range of motion, and other health issues. The loss of cervical lordosis has been implicated in poor functional outcomes after laminectomy [95], emphasizing the structural importance of maintaining cervical lordosis to support spinal cord health. This is supported by reports of a correlation between hypo-lordotic cervical curve configurations and cervicogenic pain, radiculopathies, and increased rates of degenerative discs in patients with injuries resulting from a motor vehicle accident. This is associated with several symptoms and a worse prognosis [96,97]. Understanding the significance of this curvature loss, despite attempts at trivialization, is essential for effective diagnosis and management. Framing the loss of lordosis as benign fails to address the associated physiological changes that may further complicate recovery.

## Conclusions

The application of dynamic imaging methods, such as videofluoroscopy and dynamic radiography, for the evaluation of whiplash injuries is pivotal in both clinical assessments and litigation. These methods allow healthcare providers and, if necessary, jurors to visualize the complex dynamics of cervical spine movement. Therefore, dynamic imaging serves as a powerful tool for diagnosing and validating injuries and plays a critical role in supporting claims for treatment and compensation in whiplash-related litigation.

## References

[1] Sizer PS Jr, Poorbaugh K, Phelps V (2004). Whiplash associated disorders: pathomechanics, diagnosis, and management. Pain Pract.

[2] Myrtveit SM, Skogen JC, Wenzel HG, Mykletun A (2012). Somatic symptoms beyond those generally associated with a whiplash injury are increased in self-reported chronic whiplash. A population-based cross sectional study: the Hordaland Health Study (HUSK). BMC Psychiatry.

[3] Sarrami P, Armstrong E, Naylor JM, Harris IA (2017). Factors predicting outcome in whiplash injury: a systematic meta-review of prognostic factors. J Orthop Traumatol.

[4] Kongsted A, Sorensen JS, Andersen H, Keseler B, Jensen TS, Bendix T (2008). Are early MRI findings correlated with long-lasting symptoms following whiplash injury? A prospective trial with 1-year follow-up. Eur Spine J.

[5] Laporte S, Wang D, Lecompte J (2016). An attempt of early detection of poor outcome after whiplash. Front Neurol.

[6] Yadla S, Ratliff JK, Harrop JS (2008). Whiplash: diagnosis, treatment, and associated injuries. Curr Rev Musculoskelet Med.

[7] Ivancic PC (2014). Cervical spine instability following axial compression injury: a biomechanical study. Orthop Traumatol Surg Res.

[8] Lippa L, Lippa L, Cacciola F (2017). Loss of cervical lordosis: what is the prognosis?. J Craniovertebr Junction Spine.

[9] Uchida K, Nakajima H, Sato R, Yayama T, Mwaka ES, Kobayashi S, Baba H (2009). Cervical spondylotic myelopathy associated with kyphosis or sagittal sigmoid alignment: outcome after anterior or posterior decompression. J Neurosurg Spine.

[10] Lee SH, Hyun SJ, Jain A (2020). Cervical sagittal alignment: literature review and future directions. Neurospine.

[11] Elliott JM, Dewald JP, Hornby TG, Walton DM, Parrish TB (2014). Mechanisms underlying chronic whiplash: contributions from an incomplete spinal cord injury?. Pain Med.

[12] Hauser RA, Griffiths M, Watterson A, Matias D, Rawlings BR (2025). Blurry vision—clear connection to the neck? Retrospective cross-sectional study: 145 patients with blurry vision reporting to an outpatient neck center. Preprints.

[13] Katz RS, Leavitt F, Cherny K, Small AK, Small BJ (2023). The vast majority of patients with fibromyalgia have a straight neck observed on a lateral view radiograph of the cervical spine: an aid in the diagnosis of fibromyalgia and a possible clue to the etiology. J Clin Rheumatol.

[14] Farmer PK, Snodgrass SJ, Buxton AJ, Rivett DA (2015). An investigation of cervical spinal posture in cervicogenic headache. Phys Ther.

[15] Russek LN, Block NP, Byrne E (2022). Presentation and physical therapy management of upper cervical instability in patients with symptomatic generalized joint hypermobility: international expert consensus recommendations. Front Med (Lausanne).

[16] Miura I, Horisawa S, Kawamata T, Taira T (2022). Successful treatment of focal hand dystonia after cervical whiplash injury by thalamotomy. Surg Neurol Int.

[17] Liu Y, Zhou XZ, Li N, Xu TG (2021). Relationship between cervical curvature and spinal cord drift distance after laminectomy via lateral mass screw fixation and its effect on clinical efficacy. Medicine (Baltimore).

[18] Bulut MD, Alpayci M, Şenköy E, Bora A, Yazmalar L, Yavuz A, Gülşen İ (2016). Decreased vertebral artery hemodynamics in patients with loss of cervical lordosis. Med Sci Monit.

[19] Rosa S, Baird JW (2015). The craniocervical junction: observations regarding the relationship between misalignment, obstruction of cerebrospinal fluid flow, cerebellar tonsillar ectopia, and image-guided correction. The Craniocervical Syndrome and MRI.

[20] Damadian RV, Chu D (2011). The possible role of cranio-cervical trauma and abnormal CSF hydrodynamics in the genesis of multiple sclerosis. Physiol Chem Phys Med NMR.

[21] Flanagan MF (2015). The role of the craniocervical junction in craniospinal hydrodynamics and neurodegenerative conditions. Neurol Res Int.

[22] Gay RE (1993). The curve of the cervical spine: variations and significance. J Manipulative Physiol Ther.

[23] Eck JC, Hodges SD, Humphreys SC (2001). Whiplash: a review of a commonly misunderstood injury. Am J Med.

[24] Gao K, Zhang J, Lai J (2019). Correlation between cervical lordosis and cervical disc herniation in young patients with neck pain. Medicine (Baltimore).

[25] Ames CP, Smith JS, Scheer JK (2012). Impact of spinopelvic alignment on decision making in deformity surgery in adults: a review. J Neurosurg Spine.

[26] Gehweiler JA Jr, Clark WM, Schaaf RE, Powers B, Miller MD (1979). Cervical spine trauma: the common combined conditions. Radiology.

[27] Clark WM, Gehweiler JA, Laib R (1979). Twelve significant signs of cervical spine trauma. Skelet Radiol.

[28] Kettner NW, Guebert GM (1991). The radiology of cervical spine injury. J Manipulative Physiol Ther.

[29] Deltoff MN, Kogon PL (1998). The Portable Skeletal X-ray Library. https://www.abebooks.com/9780815122449/Portable-Skeletal-X-Ray-Library-Deltoff-0815122446/plp.

[30] Rechtman AM, Boreadis Borden AG, Gershon-Cohen J (1961). The lordotic curve of the cervical spine. Clin Orthop.

[31] Fedorchuk CA, McCoy M, Lightstone DF (2016). Impact of isometric contraction of anterior cervical muscles on cervical lordosis. J Radiol Case Rep.

[32] Helliwell PS, Evans PF, Wright V (1994). The straight cervical spine: does it indicate muscle spasm?. J Bone Joint Surg Br.

[33] Kim BJ, Cho SM, Hur JW, Cha J, Kim SH (2021). Kinematics after cervical laminoplasty: risk factors for cervical kyphotic deformity after laminoplasty. Spine J.

[34] Tominaga Y, Ndu AB, Coe MP (2006). Neck ligament strength is decreased following whiplash trauma. BMC Musculoskelet Disord.

[35] Thakar S, Mohan D, Furtado SV (2014). Paraspinal muscle morphometry in cervical spondylotic myelopathy and its implications in clinicoradiological outcomes following central corpectomy: clinical article. J Neurosurg Spine.

[36] Haas JW, Oakley PA, Ferrantelli JR, Katz EA, Moustafa IM, Harrison DE (2024). Abnormal static sagittal cervical curvatures following motor vehicle collisions: a retrospective case series of 41 patients before and after a crash exposure. Diagnostics (Basel).

[37] Scher AT (1978). Ligamentous injury of the cervical spine--two radiological signs. S Afr Med J.

[38] Cramer GD (2014). The cervical region. Clinical Anatomy of the Spine, Spinal Cord, and ANS (Third Edition).

[39] Bland JH, Boushey DR (1990). Anatomy and physiology of the cervical spine. Semin Arthritis Rheum.

[40] Shekhar H, Demetriades AK (2024). Trauma of the upper cervical spine and cranio-vertebral junction in adults. Orthop Trauma.

[41] Kim DH, Vaccaro AR, Dickman CA, Cho D, Lee S, Kim I (2013). Surgical Anatomy and Techniques to the Spine. I: Surgical Anatomy and Techniques to the Spine.. Elsevier.

[42] Rajani S (2014). Is variant anatomy of atlas clinically important? A review. Basic Sci Med.

[43] Sankar WN, Wills BP, Dormans JP, Drummond DS (2006). Os odontoideum revisited: the case for a multifactorial etiology. Spine (Phila Pa 1976).

[44] Kaiser JT, Reddy V, Launico MV (2025). Anatomy, head and neck: cervical vertebrae. StatPearls [Internet].

[45] Maingard J, Campos A, Chieng R (2014). Axis (C2). Radiopaedia.org.

[46] Offiah CE, Day E (2017). The craniocervical junction: embryology, anatomy, biomechanics and imaging in blunt trauma. Insights Imaging.

[47] Hamill J, Knutzen KM, Derrick T (2006). Biomechanical Basis of Human Movement Fifth Edition. Biomechanical Basis of Human Movement.

[48] Oliver J, Middleditch A (1991). Functional Anatomy of the Spine. Functional Anatomy of the Spine.. Butterworth- Heinemann, Oxford.

[49] Schievink WI (2001). Spontaneous dissection of the carotid and vertebral arteries. N Engl J Med.

[50] Aebi M, Abumi K, Barbieri A (2011). The Craniovertebral Junction: Diagnosis, Pathology, Surgical Techniques. https://www.thieme-connect.de/products/ebooks/book/10.1055/b-002-80428.

[51] Weis S, Sonnberger M, Dunzinger A, Voglmayr E, Aichholzer M, Kleiser R, Strasser P (2019). Arterial supply of the brain. Imaging Brain Diseases.

[52] Godek P, Ruciński W (2024). Differentiating the structural and functional instability of the craniocervical junction. Healthcare (Basel).

[53] Borghei-Razavi H, Darvish O, Schick U (2014). Disabling vertigo and tinnitus caused by intrameatal compression of the anterior inferior cerebellar artery on the vestibulocochlear nerve: a case report, surgical considerations, and review of the literature. J Neurol Surg Rep.

[54] Verma R, Junewar V, Garg RK, Malhotra HS (2012). A rare case of basilar impression. BMJ Case Rep.

[55] Libreros-Jiménez HM, Manzo J, Rojas-Durán F (2024). On the cranial nerves. NeuroSci.

[56] Zimmerman EE, Misulis KE (2023). Chapter 3.4: brainstem and cranial nerves. Neurologic Localization and Diagnosis.

[57] Baquiran M, Bordoni B (2025). Anatomy, head and neck: anterior vagus nerve. StatPearls [Internet].

[58] Hauser RA, Matias D, Rawlings B (2024). The ligamentous cervical instability etiology of human disease from the forward head-facedown lifestyle: emphasis on obstruction of fluid flow into and out of the brain. Front Neurol.

[59] Hauser R (2025). Vagus nerve compression in the neck: symptoms and treatments. https://caringmedical.com/prolotherapy-news/vagus-nerve-compression-cervical-spine/.

[60] Finsterer J, Grisold W (2015). Disorders of the lower cranial nerves. J Neurosci Rural Pract.

[61] Breit S, Kupferberg A, Rogler G, Hasler G (2018). Vagus nerve as modulator of the brain-gut axis in psychiatric and inflammatory disorders. Front Psychiatry.

[62] Matsui T, Iwata M, Endo Y (2019). Effect of intensive inpatient physical therapy on whole-body indefinite symptoms in patients with whiplash-associated disorders. BMC Musculoskelet Disord.

[63] Hauser RA (2024). Hauser's laws on the ligamentous structural causes of chronic disabling symptoms of human diseases. On J Neur Br Disord.

[64] Jaggi A, Lambert SM (2010). Chapter 12 - regional complications in joint hypermobility syndrome. Hypermobility, Fibromyalgia and Chronic Pain.

[65] Okereke I, Mmerem K, Balasubramanian D (2021). The management of cervical spine injuries - a literature review. Orthop Res Rev.

[66] Ivancic PC, Ito S, Tominaga Y (2008). Whiplash causes increased laxity of cervical capsular ligament. Clin Biomech (Bristol).

[67] Hauser RA, Woldin BA (2018). Joint instability as the cause of chronic musculoskeletal pain and its successful treatment with prolotherapy. Anatomy, Posture, Prevalence, Pain, Treatment and Interventions of Musculoskeletal Disorders.

[68] Ying J, Teng H, Qian Y, Hu Y, Wen T, Ruan D, Zhu M (2019). Radiographic analysis of the correlation between ossification of the nuchal ligament and sagittal alignment and segmental stability of the cervical spine in patients with cervical spondylotic myelopathy. Acta Radiol.

[69] Fiester P, Soule E, Patel J, Jenson M, Rao D (2022). Interrelationship between craniocervical dissociation spectrum injuries and atlantoaxial instability on trauma cervical MRI examinations. Cureus.

[70] Wang W, Gao C, Ren L, Wang L, Liu C, Zeng Y (2013). Strategy of posterior decompression and extensor reconstruction in the treatment of OPLL with disease extent reaching the epistropheus. Neurosurg Q.

[71] Liao S, Jung MK, Hörnig L, Grützner PA, Kreinest M (2020). Injuries of the upper cervical spine—how can instability be identified?. Int Orthop.

[72] Gonzalez LF, Webb KM, Crawford NR, Sonntag VK (2011). Trauma to the craniovertebral junction. Craniovertebral Junction: Diagnosis, Pathology, Surgical Techniques.

[73] Dvorak J, Schneider E, Saldinger P, Rahn B (1988). Biomechanics of the craniocervical region: the alar and transverse ligaments. J Orthop Res.

[74] Mańczak M, Gasik R (2017). Cervical spine instability in the course of rheumatoid arthritis - imaging methods. Reumatologia.

[75] Daskareh M, Esmaeilian S, Rahmanipour E, Ghorbani M (2025). Evaluating the pivotal role of MRI in craniocervical junction injury diagnosis: a case report. Medicine.

[76] Zhu C, Yang HL, Im GH, Liu LM, Zhou CG, Song YM (2021). Clinical algorithm for preventing missed diagnoses of occult cervical spine instability after acute trauma: a case report. World J Clin Cases.

[77] Carter KJ, Dunham CM, Castro F, Erickson B (2011). Comparative analysis of cervical spine management in a subset of severe traumatic brain injury cases using computer simulation. PLoS One.

[78] Uhrenholt L, Gregersen M, Charles AV, Hauge EM, Nielsen E (2010). Examinations of the deceased can contribute to the understanding of whiplash injuries after traffic accidents (Article in Danish). Ugeskr Laeger.

[79] Janssen I, Sollmann N, Barz M (2021). Occult disco-ligamentous lesions of the subaxial C-spine—a comparison of preoperative imaging findings and intraoperative site inspection. Diagnostics (Basel).

[80] Katz EA, Katz SB, Freeman MD (2023). Non-surgical management of upper cervical instability via improved cervical lordosis: a case series of adult patients. J Clin Med.

[81] Yeo CG, Jeon I, Kim SW (2015). Delayed or missed diagnosis of cervical instability after traumatic injury: usefulness of dynamic flexion and extension radiographs. Korean J Spine.

[82] Mantripragada S, Kannivelu A, Peh WC (2020). Magnetic resonance imaging of cervical ligamentous anatomy and traumatic ligamentous injuries. J Med Imaging Radiat Oncol.

[83] Mahdavi A, Rasti S (2024). Dynamic flexion-extension magnetic resonance imaging of the cervical spine: an evolutionary tool for diagnosis and management of cervical spondylotic myelopathy. World Neurosurg.

[84] Mathers KS, Schneider M, Timko M (2011). Occult hypermobility of the craniocervical junction: a case report and review. J Orthop Sports Phys Ther.

[85] Dave BR, Mayi SC, Krishnan A, Kohli R, Degulmadi D, Rai RR, Dave MB (2022). A prospective study to find out the association between supine lying low back pain and retrolisthesis. Back Bone J.

[86] Karabag H, Iplikcioglu AC (2022). Simulating upright cervical lordosis in the supine position. Acta Orthop Belg.

[87] Moskopp ML, Moskopp D (2018). Automated image analysis of video fluoroscopy in the cervical spine. Ger Med Sci.

[88] Rodriquez AA, Barr KP, Burns SP (2004). Whiplash: pathophysiology, diagnosis, treatment, and prognosis. Muscle Nerve.

[89] Freeman MD, Katz EA, Rosa SL, Gatterman BG, Strömmer EMF, Leith WM (2020). Diagnostic accuracy of videofluoroscopy for symptomatic cervical spine injury following whiplash trauma. Int J Environ Res Public Health.

[90] Yoganandan N, Cusick JF, Pintar FA, Rao RD (2001). Whiplash injury determination with conventional spine imaging and cryomicrotomy. Spine (Phila Pa 1976).

[91] Bornstein BH, Greene E (2011). Jury decision making: implications for and from psychology. Curr Dir Psychol Sci.

[92] Lieberman JD, Krauss DA, Heen M, Sakiyama M (2016). The good, the bad, and the ugly: professional perceptions of jury decision‐making research practices. Behav Sci Law.

[93] Ruva CL, Guenther CC (2017). Keep your bias to yourself: how deliberating with differently biased others affects mock-jurors' guilt decisions, perceptions of the defendant, memories, and evidence interpretation. Law Hum Behav.

[94] Kaale BR, McArthur TJ, Barbosa MH, Freeman MD (2023). Post-traumatic atlanto-axial instability: a combined clinical and radiological approach for the diagnosis of pathological rotational movement in the upper cervical spine. J Clin Med.

[95] Kaptain GJ, Simmons NE, Replogle RE, Pobereskin L (2000). Incidence and outcome of kyphotic deformity following laminectomy for cervical spondylotic myelopathy. J Neurosurg.

[96] Jackson R (1964). The positive findings in alleged neck injuries. Am J Orthop.

[97] Griffiths HJ, Olson PN, Everson LI, Winemiller M (1995). Hyperextension strain or "whiplash" injuries to the cervical spine. Skeletal Radiol.

